# Plakoglobin Represses SATB1 Expression and Decreases *In Vitro* Proliferation, Migration and Invasion

**DOI:** 10.1371/journal.pone.0078388

**Published:** 2013-11-08

**Authors:** Zackie Aktary, Manijeh Pasdar

**Affiliations:** 1 Department of Oncology, University of Alberta, Edmonton, Alberta, Canada; 2 Department of Cell Biology, University of Alberta, Edmonton, Alberta, Canada; Northwestern University Feinberg School of Medicine, United States of America

## Abstract

Plakoglobin (γ-catenin) is a homolog of β-catenin with dual adhesive and signaling functions. Plakoglobin participates in cell-cell adhesion as a component of the adherens junction and desmosomes whereas its signaling function is mediated by its interactions with various intracellular protein partners. To determine the role of plakoglobin during tumorigenesis and metastasis, we expressed plakoglobin in the human tongue squamous cell carcinoma (SCC9) cells and compared the mRNA profiles of parental SCC9 cells and their plakoglobin-expressing transfectants (SCC9-PG). We observed that the mRNA levels of SATB1, the oncogenic chromatin remodeling factor, were decreased approximately 3-fold in SCC9-PG cells compared to parental SCC9 cells. Here, we showed that plakoglobin decreased levels of SATB1 mRNA and protein in SCC9-PG cells and that plakoglobin and p53 associated with the *SATB1* promoter. Plakoglobin expression also resulted in decreased *SATB1* promoter activity. These results were confirmed following plakoglobin expression in the very low plakoglobin expressing and invasive mammary carcinoma cell line MDA-MB-231 cells (MDA-231-PG). In addition, knockdown of endogenous plakoglobin in the non-invasive mammary carcinoma MCF-7 cells (MCF-7-shPG) resulted in increased SATB1 mRNA and protein. Plakoglobin expression also resulted in increased mRNA and protein levels of the metastasis suppressor Nm23-H1, a SATB1 target gene. Furthermore, the levels of various SATB1 target genes involved in tumorigenesis and metastasis were altered in MCF-7-shPG cells relative to parental MCF-7 cells. Finally, plakoglobin expression resulted in decreased *in vitro* proliferation, migration and invasion in different carcinoma cell lines. Together with the results of our previous studies, the data suggests that plakoglobin suppresses tumorigenesis and metastasis through the regulation of genes involved in these processes.

## Introduction

Metastasis is a multi-step process that begins when tumor cells acquire the ability to degrade the basement membrane and move from the primary site of tumor formation to distant sites throughout the body, culminating in the formation of secondary tumors at these new sites. It is the formation of these secondary tumors that is the major cause of cancer-related deaths. In epithelial tissues, the abnormal proliferation, migration and invasion of constituent cells are limited by intercellular adhesive complexes, which tether neighboring cells to one another and maintain normal tissue architecture and function [1]–[5].

The main adhesive complexes in epithelia are the cadherin-based adherens junction and desmosomes [6]–[7]. Cadherins are single-pass transmembrane glycoproteins that make homotypic extracellular interactions with cadherin proteins on neighboring cells and intracellularly interact with catenin proteins [5]. At the adherens junction, E-cadherin interacts with either β-catenin or γ-catenin (plakoglobin), which then interact with α-catenin, an actin binding protein, which tethers the cadherin-catenin complex to the actin cytoskeleton [5]. Similarly, at the desmosome, the desmosomal cadherins (desmocollins and desmogleins) are tethered to the intermediate filament cytoskeleton through interactions with plakoglobin and desmoplakin [6]–[7].

β-catenin and plakoglobin are structural and functional homologs and members of the armadillo family of proteins with dual functions in cell-cell adhesion and cell signaling [8]–[10]. Both proteins interact with E-cadherin, Axin and APC and both are involved in the Wnt signaling pathway through their interactions with the TCF/LEF transcription factors. Despite their structural similarities and common interacting partners, β-catenin and plakoglobin appear to have different signaling activities and regulate tumorigenesis in opposite manners. While β-catenin-TCF/LEF complexes are transcriptionally active, several studies have demonstrated that plakoglobin-TCF complexes are inefficient in binding to DNA [11]–[13]. Conversely, plakoglobin, but not β-catenin, interacts with p53 and regulates gene expression independent of TCF [14]. Furthermore, β-catenin has well-documented oncogenic signaling activities as the terminal component of the Wnt signaling pathway, whereas plakoglobin has typically been associated with tumor/metastasis suppressor activities [14]–[22].

To determine the role of plakoglobin in tumorigenesis and metastasis, we previously expressed physiological levels of plakoglobin in the plakoglobin-null SCC9 cell line, a human squamous cell carcinoma cell line derived from the tongue. Plakoglobin expression in SCC9 cells (SCC9-PG) resulted in a mesenchymal (transformed)-to-epidermoid (normal) phenotypic transition that was concurrent with the increased levels of N-cadherin, decreased levels of β-catenin and the formation of desmosomes [15]. We subsequently performed proteomic and transcription microarray experiments to identify potential genes and proteins whose levels were differentially expressed following plakoglobin expression. These studies identified several tumor and metastasis suppressors and oncogenes whose levels were increased and decreased, respectively, in SCC9-PG cells. Among these differentially expressed genes was the global regulator of gene expression, Special AT-Rich Sequence Binding Protein 1 (SATB1).

SATB1 was initially identified as a DNA-binding protein that was highly expressed in the thymus [23]–[24]. This protein was shown to have a high affinity for binding to base-unpairing regions (BURs), which are genomic DNA sequences with high unfolding potential, containing clusters of sequences (approximately 20–40 base pairs long) with a bias in G and C distribution, with one DNA strand contains only A, T and C residues [23], [25]–[27]. Importantly, since BUR sequences are thought to be found all throughout the genome and since SATB1 demonstrated a specificity for these BUR sequences, it became evident that SATB1 could, through its interactions with different BUR sequences in different gene promoters, cause the looping of chromatin [26], [28]–[30]. These chromatin loops could, in turn, potentially result in the close physical proximity and coordinated regulation of genes that would otherwise remain silent. In addition to forming these chromatin loops, SATB1 was shown to recruit different chromatin remodeling enzymes to the gene loci close to the BURs and as a result altered gene expression [31]–[34].

SATB1, through the regulation of gene expression, has been shown to promote *in vitro* tumorigenesis and metastasis in various cell lines, including breast, lung, ovarian, colorectal, liver, laryngeal, glioma and melanoma [34]–[42]. Specifically, SATB1 has been shown to induce the expression of tumor and metastasis-promoting genes while suppressing the expression of metastasis suppressor genes [34], [39], [43]–[44]. Interestingly, our microarray experiments showed that SATB1 expression was decreased 3-fold in SCC9-PG cells compared to SCC9 cells [14], suggesting that plakoglobin may play a role in regulating the *SATB1* gene and thus, may have an effect on a the expression of a wide range of tumorigenesis and metastasis associated genes.

In the present study, we examined the role of plakoglobin in regulating the expression of genes involved in tumorigenesis and metastasis. We showed that plakoglobin, in coordination with p53, associated with the *SATB1* promoter and downregulated its expression. Decreased levels of SATB1 mRNA were accompanied by its decreased protein levels in squamous and mammary epithelial cell lines expressing plakoglobin. Furthermore, plakoglobin expression led to an increase and a decrease in the protein levels of a subset of SATB1 repressed and activated target genes, respectively. Concurrent with these transcriptional changes, plakoglobin expression resulted in decreased cell growth and *in vitro* migration and invasion. Taken together, our data suggests that plakoglobin suppresses tumorigenesis and metastasis (at least *in vitro*) through the regulation of genes involved in these processes.

## Materials and Methods

### Cell culture and conditions

All tissue culture reagents were purchased from Invitrogen (Burlington, Canada) unless stated otherwise. SCC9, SCC9-PG, MDA-231, MDA-231-PG, MCF-7 and MCF-7-shPG cells have all been described previously [14]–[15], [45].

### Construction of SATB1-luciferase reporter constructs

The *SATB1* promoter was cloned from SCC9 genomic DNA by PCR and ligated into the pBV-Luc vector at KpnI and SacI sites, respectively. The primer sequences used for the cloning reaction were CAGTggtaccGCCA-GGGCGACTCTAGAG (forward, starting at base pair 14 in the *SATB1* gene) and AGCTgagctcCACTTCAAAACTTGACAGCACATA (reverse, ending at base pair 1222 in the *SATB1* gene). The constructed luciferase plasmid was then used for reporter assays.

### RNA isolation and RT-PCR

RNA was isolated from 150 mm confluent cultures using the RNeasy Plus Mini Kit (QIAGEN, Valencia, CA) according to the manufacturer's protocol. Following isolation, RNA was pre-treated with RNase-free DNaseI and reverse transcribed using the RevertAid H Minus First Strand cDNA Synthesis Kit (Fermentas, Burlington, ON, Canada). Polymerase chain reaction (PCR) was performed (Fermentas, Burlington, ON, Canada) on the amplified cDNA. The sequences of the primers used are outlined in Table 1. RT-PCR products were resolved on 2% agarose gels and visualized by ethidium bromide staining. qRT-PCR was performed using PerfeCta SYBR Green FastMix reagent (Quanta Biosciences) as per the manufacturer's instructions.

**Table 1:** Primer sequences and PCR conditions for reverse transcribed genes.

Gene	Primers	Size (bp)	Annealing temperature	Ref.
**RT-PCR**				
ABL1	**Sense**: 5′- GTATTTCACAGAGCACGCCT-3′; **Antisense**: 5′- GAGGTGATGTGCTGTAAGA-3′	-	60°C	[34]
BRMS1	**Sense**: 5′-GGAAGAAGGCACCTCTGGTTT-3′; **Antisense**: 5′- GCTGCCCTAGCCTTTTTGATG-3′	-	60°C	[34]
CLDN1	**Sense**: 5′- CCCCAGTGGAGGATTTACTCCTA-3′; **Antisense**: 5′- GCAATGTGCTGCTCAGATTCA-3′	-	60°C	[34]
ERBB2	**Sense**: 5′- AGGGCAGTTACCAGTGCCAATATC-3′; **Antisense**: 5′- TCCAGAGTCTCAAACACTTGGAGC-3′	-	60°C	[34]
KISS1	**Sense**: 5′- CCATTAGAAAAGGTGGCCTCTGT-3′; **Antisense**: 5′- AGGAGGCCCAGGGATTCTAG-3′	-	60°C	[34]
MMP3	**Sense**: 5′- CGATGCAGCCATTTCTGATAAG-3′; **Antisense**: 5′- GCCTGGCTCCATGGAATTT-3′	-	60°C	[34]
SNAI1	**Sense**: 5′- CCCCAATCGGAAGCCTAACT-3′; **Antisense**: 5′- GGACAGAGTCCCAGATGAGCAT-3′	-	60°C	[34]
NME1	**Sense**: 5′-CGCAGTTCAAACCTAAGCAGCAGCTGG-3′; **Antisense**: 5′- GATCCAGTTCTGAGCACAGCTCG-3′	483	60°C	[93]
NME2	**Sense**: 5′-TGACCTGAAAGACCGACCAT-3′; **Antisense**: 5′-GAATGATGTTCCTGCCAACC-3′	193	55°C	[94]
SATB1	**Sense**: 5′- TGCAAAGGTTGCAGCAACCAAAAGC-3′; **Antisense**: 5′- AACATGGATAATGTGGGGCGGCCT-3′	156	60°C	[34]
GAPDH	**Sense**: 5′-GAAGGTGAAGGTCGGAGTC-3′; **Antisense**: 5′-GAAGATGGTGATGGGATTTC-3′	220	60°C	[95]
**ChIP**				
NME1	**Sense**: 5′-CAACTGTGAGCGTACCTTCAT-3′; **Antisense**: 5′-AACAAGGCGGAATCCTTTCTG-3′	102	53.6°C	-
SATB1	**Sense**: 5′-GATCATTTGAACGAGGCAACTCA-3′; **Antisense**: 5′-CCTGCATTTTTGCACCTGTACT-3′	157	53.6°C	-

For all primers, pre-denaturation was done at 95°C for 5 minutes. This was followed by 35 cycles of denaturation at 95°C for 30 seconds, annealing for 45 seconds, and extension at 72°C for 45 seconds.

### Antibodies

A list of antibodies and their respective dilutions in specific assays is presented in Table 2.

**Table 2:** Antibodies and their respective dilutions in specific assays.

		Assays			
Antibodies	Species	WB	ChIP	IF	Source
β-Actin	Mouse	1:2000	-	-	Sigma, A-5441
Anti-mouse IgG	Goat	-	1:2000	-	Sigma, M-5899
BrdU	Mouse	-	-	1:300	Sigma, B-5002
BRMS1	Mouse	1:200	-	-	Santa Cruz, sc-101219
c-Abl	Rabbit	1:1000	-	-	Santa Cruz, sc-131
Claudin-1	Mouse	1:500	-	-	Santa Cruz, sc-137121
ErbB2	Rabbit	1:1000	-	-	Upstate, 06–562
Kiss1	Rabbit	1:500	-	-	Santa Cruz, sc-15400
MMP3	Mouse	1:100	-	-	Calbiochem, Ab-1
Nm23-H1/H2	Rabbit	1:200	-	-	Chemicon, CBL-446
p53	Mouse	-	1:100	-	Santa Cruz, sc-126
SATB1	Rabbit	1:1000	-	-	Cell Signaling, L745
Snail	Rabbit	1:2000	-	-	Abcam, ab17732
Plakoglobin	Mouse	1:500	1:100	-	Transduction Laboratories, 610254
**2° Antibodies**					
Anti-mouse HRP, Light Chain specific	Goat	1:5000	-	-	Jackson, 115-005-174
Anti-rabbit HRP, Light Chain specific	Goat	1:5000	-	-	Jackson, 211-002-177
Alexa Fluor 488	Goat	-	-	1:1500	Molecular Probes, A11035

### Preparation of total cell extracts and Western blotting

Confluent 150 mm culture dishes were washed twice with cold phosphate-buffered saline (PBS), solubilized in hot SDS sample buffer (10 mM Tris-HCl pH 6.8, 2% (w/v) SDS, 50 mM dithiothreitol (DTT), 2 mM EDTA, 0.5 mM PMSF) and boiled for 10 minutes. Protein determination was done using Bradford (Pierce) assays according to the manufacturer's instructions. Seventy-five micrograms of total cellular protein were resolved by SDS-PAGE, transferred to nitrocellulose membranes and processed for immunoblotting and developed by standard ECL (Perkin Elmer, Woodbridge, Canada) procedures.

### Chromatin immunoprecipitation

Chromatin immunoprecipitation (ChIP) experiments were performed as previously described [14], [46]. Confluent 150 mm cultures were trypsinized and 2×10^7^ cells pelleted by centrifugation at 1500×g for 10 minutes. The cell pellets were then resuspended in growth media to which formaldehyde (Fisher) was added to a final concentration of 1% and incubated at room temperature for 10 minutes. To stop fixation, glycine was added to a final concentration of 125 mM. The cell suspension was then centrifuged at 1500×g at 4°C for 10 minutes. The resulting cell pellets were then washed twice with PBS containing 1 μg/ml aprotinin and leupeptin and 1 mM PMSF, after which they were resuspended in cell lysis buffer (10 mM HEPES pH 7.9, 10 mM KCl, 0.1 mM EDTA, 0.1 mM EGTA, 1 mM DTT and 0.49 mM PMSF) and incubated on ice for 15 minutes. NP-40 was then added (final concentration of 0.6%) after which the samples were vortexed for 10 seconds at high speed and subsequently centrifuged at 18,000×g for 30 seconds. The resulting pellets were then resuspended in sonication buffer (1% SDS, 10 mM EDTA, 50 mM Tris pH 8, 0.49 mM DTT and 0.02 μg/ml aprotinin and leupeptin) and left on ice for 10 minutes. The samples were then sonicated (Branson Sonifier 450) for 1 minute at 20% output for a total of four times.

The sonicated chromatin samples were then diluted ten-fold in chromatin dilution buffer (0.01% SDS, 1.1% Triton X-100, 1.2 mM EDTA, 16.7 mM Tris pH 8, 167 mM NaCl) after which 50 μl was removed (Input). Forty μl Protein A/G agarose beads (Calbiochem) were added and the samples were pre-cleaned on a rocker-rotator at 4°C for 2 hours. Following incubation, the samples were centrifuged briefly and the resulting supernatant (pre-cleaned chromatin) was split into equal aliquots and processed for immunoprecipitation. Each aliquot was incubated with 5 μg antibodies and 40 μl pre-cleaned (by overnight incubation with 4 μg Salmon Sperm DNA and BSA) Protein A/G agarose beads overnight at 4°C on a rocker-rotator.

Following immunoprecipitation, the samples were centrifuged for 10 minutes at 420×g at 4°C, after which the resulting supernatants were removed. The beads were then subjected to six 5 minute washes in each of the four following wash buffers: W1 (1% SDS, 1% Triton X-100, 2 mM Tris pH 8, 167 mM NaCl), W2 (0.1% SDS, 1% Triton X-100, 2 mM EDTA, 20 mM Tris pH 8, 500 mM NaCl), W3 (250 mM LiCl, 1% NP-40, 1% sodium deoxycholate, 10 mM Tris pH 8, 1 mM EDTA) and W4 (10 mM Tris pH 8 and 1 mM EDTA). Following the washes, the protein-DNA complexes were eluted off the beads by incubation in elution buffer (1% SDS and 50 mM NaHCO_3_) for 15 minutes at room temperature on a rocker-rotator. Following elution, 1 μg RNase and NaCl (final concentration 300 mM) were added to the samples, which were then incubated at 65°C for 4 hours. Next, Tris (pH 6.8) and EDTA (final concentrations of 40 mM and 10 mM, respectively) and 4 μg proteinase K were added to the samples and the samples were incubated at 45°C for 2 hours. The samples were then purified using a PCR Purification Kit (QIAGEN, Valencia, CA) and processed for PCR.

### Luciferase reporter assay

Confluent 35 mm cultures were transfected with 4 μg of various luciferase reporter plasmids. *SATB1* promoter activity was analyzed by using a reporter construct downstream of the full *SATB1* promoter [47] together with 1 μg of a plasmid encoding β-galactosidase. Forty-eight hours post-transfection, luciferase and β-galactosidase activities were measured. To assess activity from the *NME1* promoter, cells were transfected with a reporter plasmid downstream of the *NME1* promoter, a kind gift of Dr. Shimian Qu, Vanderbilt University, Nashville, TN, USA [48]. Each experiment was repeated at least 3 times and the mean with standard deviation was calculated. Statistical analysis was performed using a Student's t-test.

### Cell growth and proliferation assays

To measure growth, 5×10^4^ cells for each cell line were plated in triplicate in a 24-well plate. At 3, 5 and 7 days after plating, cultures were trypsinized and the cells were counted. Cell proliferation was assessed by performing BrdU incorporation experiments. For each cell line, 5×10^4^ cells were plated on glass coverslips and allowed to proliferate for 6 days at which times they were incubated with BrdU (100 μM; Sigma B-5002) for 24 hours. To detect BrdU-labeled cells, coverslips were first prefixed by the addition of 3.7% formaldehyde directly to the culture media at a 1∶1 ratio (volume). Coverslips were then rinsed, fixed with 3.7% formaldehyde for 15 minutes and permeabilized with 0.5% Triton X-100 for 5 minutes. Coverslips were then washed with PBS and incubated in 2N HCl for 1 hour at room temperature followed by two 5 minute washes with 100 mM sodium borate (pH 8.5). Subsequently, coverslips were blocked for 1 hour with 4.0% goat serum and 50 mM NH_4_Cl_4_ in PBS containing 0.2% BSA (PBS-BSA) and incubated with a mouse monoclonal anti-BrdU antibody (1∶300; BD 347583) for 1 hour at room temperature followed by a 20 minute incubation with Alexa Fluor 488 (Molecular Probes, A11035) secondary antibodies. All antibodies were diluted in PBS-BSA. Nuclei were counterstained for 5 min with DRAQ5 (1∶40,000 in PBS; Biostatus). Coverslips were mounted in elvanol containing 0.2% (w/v) paraphenylene diamine (PPD) and viewed using a 63X objective of an LSM510 META (Zeiss) laser scanning confocal microscope.

### Transwell cell migration and invasion assays

For cell migration assays, 2×10^5^ cells were resuspended in 0.5 ml serum-free media containing 0.1% BSA and plated in the upper chamber of transwells (3 μm pore, 6.5 μm diameter; BD Biosciences, MD, USA). Normal media containing 10% FBS (0.75 ml) was added to the lower chamber. Cultures were incubated at 37°C for 12 or 48 hours to allow cell migration at which time the inserts were removed from the chambers, gently submerged in PBS to remove the unattached cells, fixed and stained using Diff Quick (IHC World, MD, USA). Following staining, membranes were cut, mounted on slides using permount (Fisher, Canada), viewed under an inverted microscope using a 20X objective and photographed. The migrated cells on the underside of the membrane were counted in five random fields for each transwell filter from the photographs.

Matrigel invasion assays were performed according to the manufacturer's protocol (BD Bioscience). For each cell line, 5×10^5^ cells in 0.6 ml serum-free media containing 0.1% BSA were plated in the top compartment of Matrigel-coated invasion chambers (8 μm pore membrane). Fibroblast conditioned media (0.75 ml) was added to the bottom chambers and plates were incubated at 37°C in 5% CO_2_. Forty-eight hours later, the membranes were recovered, fixed, stained with Diff Quick, viewed under an inverted microscope using a 20X objective and photographed. The invaded cells were counted in five random fields for each membrane.

Each assay was repeated 3 independent times. The numbers of migrated/invaded cells were calculated using the ImageJ Cell Counter program and averaged.

## Results

### Plakoglobin regulates SATB1 expression

**Figure 1:**
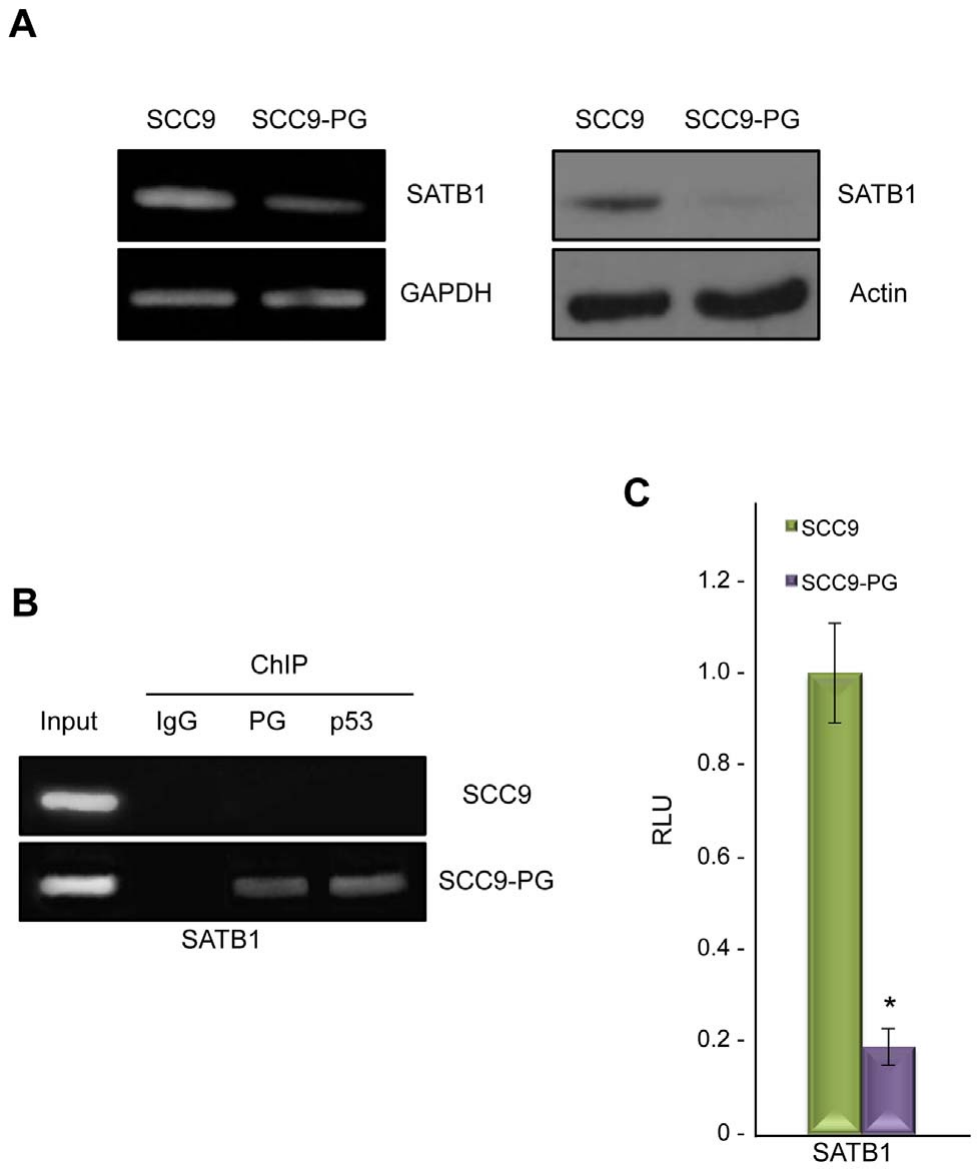
Plakoglobin associates with and suppresses the *SATB1* promoter in SCC9-PG cells. **A.** (Left) Total cellular RNA was isolated from SCC9 and SCC9-PG cells, reverse transcribed and processed for PCR using primers specific to SATB1 and GAPDH. (Right) Equal amounts of total cellular proteins from SCC9 and SCC9-PG cells were resolved by SDS-PAGE and processed for immunoblotting with antibodies to SATB1 and Actin. **B.** SCC9 and SCC9-PG cells were formaldehyde fixed and processed for chromatin immunoprecipitation. Following sonication, extracts were immunoprecipitated using control IgG, plakoglobin and p53 antibodies. Following extensive washes, immunoprecipitated DNA was separated from the immune complexes and purified using standard DNA purification protocols. The purified DNA was then processed for PCR using SATB1 primers. As a positive control, total cellular DNA (Input) was amplified using the same primers. **C.** SCC9 and SCC9-PG cells were transfected with luciferase reporter constructs under the control of a 1.2 kb sequence of the *SATB1* promoter. Luciferase activities were measured 48 hours post-transfection. The levels of luciferase activities from the vector and SATB1 reporter constructs were determined from a minimum of three independent transfections and normalized for transfection efficiency by co-transfection with a β-galactosidase expression vector. The *SATB1* promoter activity was normalized to the corresponding vector activity for each cell line and then normalized to SCC9 (*p<0.01). PG, plakoglobin; RLU, Relative Light Units.

We previously observed that plakoglobin expression in the plakoglobin-null SCC9 squamous carcinoma cell line (SCC9-PG) resulted in a mesenchymal-to-epidermoid phenotypic transition that coincided with the formation of stable cell-cell junctions [15]. Further characterization of SCC9-PG cells using transcription microarray experiments revealed that the expression of the *SATB1* gene was decreased over 3-fold in SCC9 cells following plakoglobin expression [14]. To confirm this observation, we first performed RT-PCR experiments and observed a notable decrease in SATB1 mRNA in SCC9-PG cells compared to SCC9 cells (Fig. 1A, left). In agreement with this result, western blot analysis revealed that while SATB1 protein was expressed in SCC9 cells, its levels were significantly decreased and barely detectable in SCC9-PG cells (Fig. 1A, right).

To determine whether plakoglobin regulates the *SATB1* gene, we performed chromatin immunoprecipitation (ChIP) experiments using plakoglobin antibodies and nuclear extracts from SCC9 and SCC9-PG cells. The isolated DNA was then processed for PCR using primers specific to the *SATB1* promoter (Table 1). These experiments showed that plakoglobin associated with the *SATB1* promoter in SCC9-PG cells, but not in SCC9 cells (Fig. 1B). ChIP experiments using control IgG antibodies produced negative results. Since plakoglobin interacts with and regulates gene expression in conjunction with p53 [14], we also performed the ChIP experiments using p53 antibodies, which demonstrated that while p53 associated with the *SATB1* promoter in SCC9-PG cells, this association was absent in SCC9 cells (Fig. 1B).

The association of plakoglobin and p53 with the *SATB1* promoter and the decreased levels of SATB1 mRNA and protein in SCC9-PG cells suggested that plakoglobin and p53 may function as negative regulators of the *SATB1* promoter. To test this hypothesis, luciferase reporter assays were conducted using luciferase reporter constructs downstream of a 1.2 kb *SATB1* promoter fragment [47]. Consistent with the role of plakoglobin in the negative regulation of the *SATB1* promoter, the luciferase activity of the reporter constructs was significantly decreased (over 5-fold) in SCC9-PG cells compared to SCC9 cells (Fig. 1C).

### Plakoglobin regulates SATB1 in mammary epithelial cell lines

**Figure 2:**
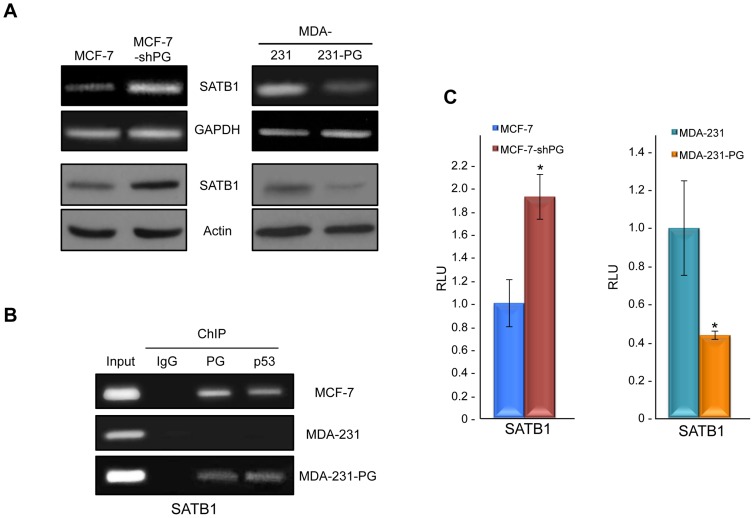
Plakoglobin suppresses *SATB1* in mammary epithelial cell lines. **A.** (Top) Total cellular RNA was isolated from MCF-7, MCF-7-shPG, MDA-231 and MDA-231-PG cells, reverse transcribed and processed for PCR using primers specific to SATB1 and GAPDH. (Bottom) Equal amounts of total cellular proteins from these cells were resolved by SDS-PAGE and processed for immunoblotting with antibodies to SATB1 and Actin.**B.** MCF-7, MDA-231 and MDA-231-PG cells were formaldehyde fixed and processed for chromatin immunoprecipitation as described in Fig. 1B. The purified DNA was then processed for PCR using SATB1 primers. As a positive control, total cellular DNA (Input) was amplified using the same primers. **C.** MCF-7, MCF-7-shPG, MDA-231 and MDA-231-PG cells were transfected with luciferase reporter constructs and processed as described in Fig. 1C. The *SATB1* promoter activity was normalized to the corresponding vector activity for each cell line and then normalized to MDA-231 or MCF-7, respectively (*p<0.01). PG, plakoglobin; RLU, Relative Light Units.

In addition to SCC9 cells, we also examined the role of plakoglobin in regulating SATB1 in MCF-7 and MDA-MB-231 (MDA-231) mammary epithelial cell lines, since SATB1 has previously been shown to play a major role in the regulation of breast cancer progression and metastasis [34]. As such, we set out to determine whether the results from SCC9-PG could be extended to breast cancer cell lines. To do so, we took two approaches: first, we knocked down plakoglobin in MCF-7 cells (MCF-7-shPG), which are non-invasive and express considerable levels of wild-type plakoglobin [14], [19], [49] and second, we expressed plakoglobin in MDA-231 cells (MDA-231-PG), which are highly invasive and express very low levels of endogenous plakoglobin [49]. RT-PCR and western blot experiments showed that knockdown of plakoglobin in MCF-7 cells resulted in increased levels of both SATB1 mRNA and protein. In contrast, plakoglobin expression in MDA-231 cells resulted in a decrease in both SATB1 mRNA and protein, although SATB1 protein was still detectable in MDA-231-PG cells (Fig. 2A).

ChIP experiments showed that similar to SCC9-PG cells, both plakoglobin and p53 associated with the *SATB1* promoter in MCF-7 cells. Furthermore, both proteins associated with the *SATB1* promoter in MDA-231-PG cells, but not MDA-231 cells (Fig. 2B). To further demonstrate that plakoglobin and p53 negatively regulate the *SATB1* promoter, we performed luciferase assay experiments using the SATB1-luciferase reporter constructs in MCF-7, MCF-7-shPG, MDA-231 and MDA-231-PG cells. The results of these experiments were consistent with those from SCC9-PG cells: luciferase activity in MDA-231-PG cells was decreased (over 2-fold) compared to MDA-231 cells, whereas activity in MCF-7-shPG cells was induced (approximately 2-fold) compared to MCF-7 cells (Fig. 2C). Taken together, the results from these experiments suggest that plakoglobin and p53 negatively regulate *SATB1* expression.

### Plakoglobin associates with and activates the NME1 promoter

**Figure 3:**
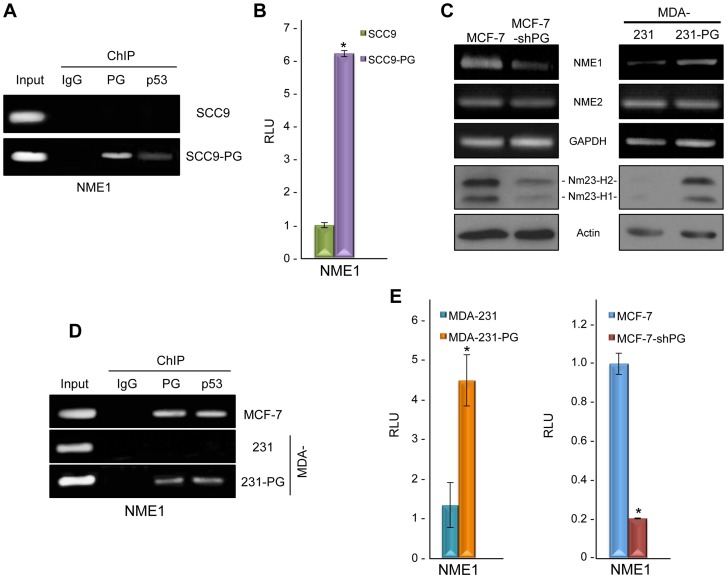
Plakoglobin associates with and activates *NME1*. **A.** SCC9 and SCC9-PG cells were processed for chromatin immunoprecipitation using control IgG, plakoglobin and p53 antibodies as described in Fig. 1B. The purified DNA was then processed for PCR using NME1 primers. As a positive control, total cellular DNA (Input) was amplified using the same primers. **B.** SCC9 and SCC9-PG cells were transfected with luciferase reporter constructs under the control of a 2 kb sequence of the *NME1* promoter. Luciferase activities were measured 48 hours post-transfection. The levels of luciferase activities from the vector and NME1 reporter constructs were determined from a minimum of three independent transfections and normalized for transfection efficiency by co-transfection with a β-galactosidase expression vector. The NME1 promoter activity was normalized to the corresponding vector activity for each cell line and then normalized to SCC9 (*p<0.01). PG, plakoglobin; RLU, Relative Light Units. **C.** (Top) Total cellular RNA was isolated from MCF-7, MCF-7-shPG, MDA-231 and MDA-231-PG cells, reverse transcribed and processed for PCR using primers specific to NME1, NME2 and GAPDH. (Bottom) Equal amounts of total cellular proteins from these cells were resolved by SDS-PAGE and processed for immunoblotting with antibodies to Nm23-H1, -H2 and Actin. **D.** MCF-7, MDA-231 and MDA-231-PG cells were processed for chromatin immunoprecipitation using control IgG, plakoglobin and p53 antibodies and the purified DNA processed for PCR using NME1 primers. As a positive control, total cellular DNA (Input) was amplified using the same primers. **E.** MCF-7, MCF-7-shPG, MDA-231 and MDA-231-PG cells were transfected with luciferase reporter constructs as described in Fig. 3B. The *NME1* promoter activity was normalized to the corresponding vector activity for each cell line and then normalized to MDA-231 or MCF-7, respectively (*p<0.01). PG, plakoglobin; RLU, Relative Light Units.

It has been suggested that the metastasis suppressor Nm23-H1 is a potential target of SATB1 [34]. Furthermore, we previously showed that plakoglobin expression in SCC9 cells resulted in increased Nm23-H1 and -H2 protein levels as well as increased Nm23-H1 (*NME1*), but not Nm23-H2 (*NME2*) gene expression [19]. Therefore, we set out to determine if the increased expression of *NME1* in SCC9-PG cells were simply due to decreased SATB1 levels or whether plakoglobin actively promoted the expression of *NME1*. In order to do so, we performed ChIP experiments using plakoglobin antibodies and primers specific to the *NME1* promoter. Plakoglobin associated with the *NME1* promoter in SCC9-PG cells, but not SCC9 cells (Fig. 3A). Similar ChIP experiments were performed using p53 antibodies, which demonstrated that while p53 associated with the *NME1* promoter in SCC9-PG cells, this association was absent in SCC9 cells (Fig. 3A). ChIP experiments using control IgG antibodies produced negative results.

To confirm the role of plakoglobin in the regulation of *NME1* expression, luciferase assays were done using luciferase reporter constructs downstream of a 2 kb *NME1* promoter fragment [48]. In these experiments, luciferase activity was induced approximately 6-fold in SCC9-PG cells compared to SCC9 cells (Fig. 3B), demonstrating that plakoglobin expression resulted in increased *NME1* promoter activity. Taken together, these data suggest that plakoglobin actively regulates the *NME1* gene through its associations with the *NME1* promoter, while it also downregulates SATB1 levels, which may in turn result in increased *NME1* expression.

### Plakoglobin regulates NME1 in mammary epithelial cell lines

We subsequently performed RT-PCR and western blot experiments to examine the levels of Nm23-H1 mRNA and protein in the mammary epithelial cell lines to confirm that plakoglobin-mediated regulation of *NME1* was not specific to squamous cell lines. Knockdown of plakoglobin in MCF-7 cells resulted in a notable decrease in Nm23-H1 mRNA, which was accompanied by a corresponding decrease in the levels of Nm23-H1 and -H2 protein (Fig. 3C). In contrast, the levels of both Nm23-H1 mRNA and protein were increased considerably in MDA-231-PG cells compared to parental MDA-231 cells (Fig. 3C). We also performed the RT-PCR experiments using primers specific to the Nm23-H2 (*NME2*) gene and observed that plakoglobin expression had no effect on *NME2* expression, since the levels of Nm23-H2 mRNA were not different between MCF-7 and MCF-7-shPG and MDA-231 and MDA-231-PG cells, respectively (Fig. 3C). These results were consistent with the lack of *NME2* induction following plakoglobin expression in SCC9-PG cells [19].

Next, ChIP experiments were conducted with chromatin from MCF-7, MDA-231 and MDA-231-PG cells using plakoglobin and p53 antibodies. The results from these experiments showed that plakoglobin and p53 associated with the *NME1* promoter in both MCF-7 and MDA-231-PG cells, but not MDA-231 cells (Fig. 3D). In addition, luciferase reporter assays using these cell lines were performed to determine the role of plakoglobin in the regulation of the *NME1* promoter. While minimal luciferase activity was observed in MDA-231 cells, promoter activity was induced over 3-fold in MDA-231-PG cells (compared to parental MDA-231 cells; Fig. 3E). In contrast, luciferase activity was decreased by ∼5-fold in MCF-7-shPG cells compared to MCF-7 cells (Fig. 3E). Taken together, these results suggest that plakoglobin and p53 positively regulate the expression of the *NME1* gene and that plakoglobin expression has no effect on the *NME2* gene.

### Changes in SATB1 target gene expression in response to plakoglobin levels

**Figure 4:**
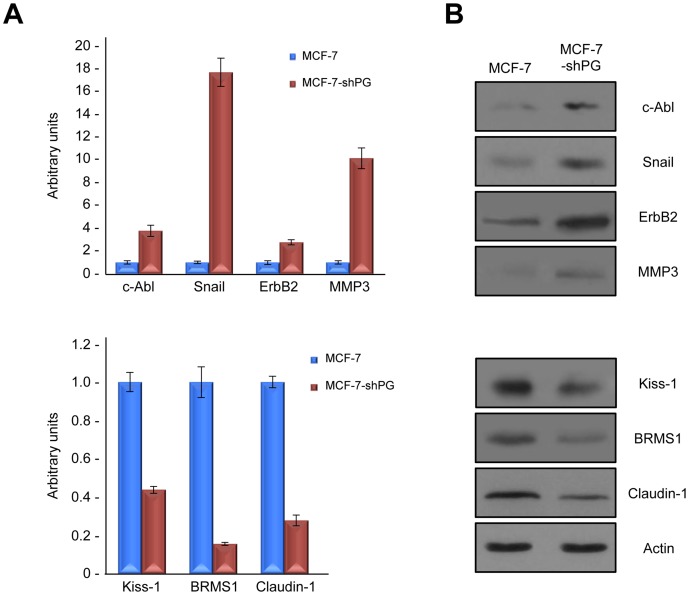
Plakoglobin knockdown changes the levels of SATB1 target genes. **A.** Total cellular RNA was isolated from MCF-7 and MCF-7-shPG cells, reverse transcribed and processed for PCR using primers specific to SATB1 target genes c-Abl, MMP3, ErbB2, Snail, BRMS1, Kiss1 and Claudin-1. **B.** Equal amounts of total cellular proteins from these cells were resolved by SDS-PAGE and processed for immunoblotting with antibodies to c-Abl, MMP3, ErbB2, Snail, BRMS1, Kiss1 and Claudin-1. PG, plakoglobin.

Since SATB1 is a major global regulator of gene expression, we argued that the alteration in SATB1 levels based on plakoglobin expression would result in alterations in the expression of various SATB1 target genes in addition to Nm23. More specifically, we focused on a select number of SATB1 target genes that are known to participate in tumorigenesis and metastasis (e.g. tumor/metastasis suppressors BRMS1, Kiss1, Claudin-1; tumor/metastasis promoters c-Abl, MMP3, ErbB2 and Snail). We performed qRT-PCR experiments and observed that the levels of c-Abl, Snail, ErbB2 and MMP3 mRNA were all increased in MCF-7-shPG cells, compared to MCF-7 cells. Consistent with the increased mRNA levels, western blot experiments showed that protein levels of these tumor/metastasis promoters were also increased in MCF-7-shPG cells (Fig. 4A-B, top). Furthermore, the mRNA and protein levels of tumor/metastasis suppressors BRMS1, Kiss1 and Claudin-1 were decreased in MCF-7-shPG cells relative to MCF-7 cells (Fig. 4A-B, bottom).

### Plakoglobin suppresses cancer cell growth, migration and invasion

**Figure 5:**
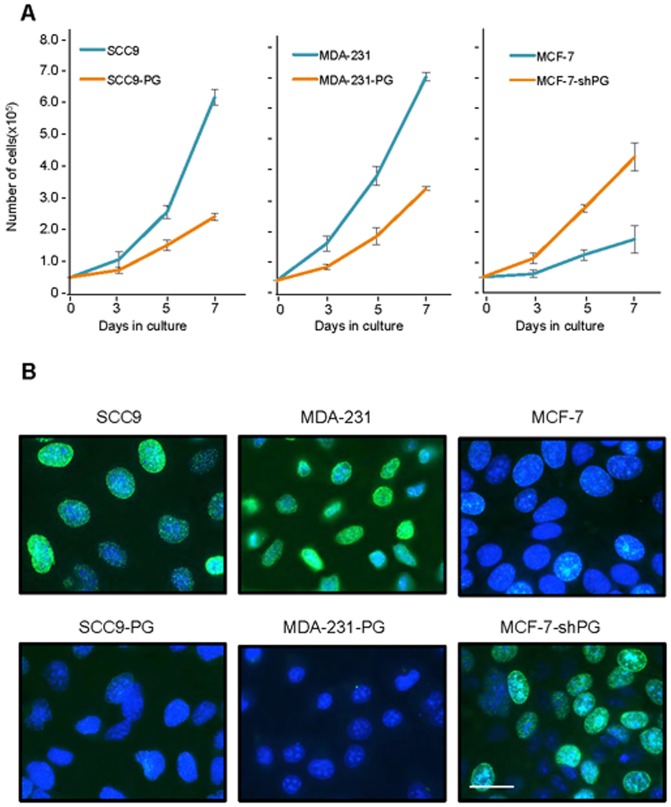
Plakoglobin decreases *in vitro* cell growth and proliferation. **A.** Replicate cultures of SCC9, SCC9-PG, MDA-231, -231-PG, MCF-7 and MCF-7-shPG cells were established at single cell density and cells were counted at 3, 5 and 7 days. Each time point represents the average of three independent experiments. The absence of error bars at some time points is due to the small differences among the experiments. **B.** SCC9, SCC9-PG, MDA-231, -231-PG, MCF-7 and MCF-7-shPG cells were plated on glass coverslips and allowed to grow for 6 days at which time BrdU was added to the cell cultures for 24 hours. BrdU incorporation was then assessed by immunofluorescence staining using BrdU antibodies. Nuclei were countersatined with DRAQ5 and cells viewed using a 63X objective of an LSM510 META (Zeiss) laser scanning confocal microscope. Bar, 20 μm.

The results so far suggested that plakoglobin plays a role in promoting the expression of various genes involved in suppressing tumor growth, migration and invasion and suppressing the expression of genes that promote these processes. In order to determine whether plakoglobin's regulation of gene expression results in a biologically discernable effect on the *in vitro* growth and the migratory and invasive properties of cells, MCF-7 and MCF-7-shPG cells we processed for growth, migration and invasion assays as described in Materials and Methods. The results of the growth assay showed a significant increase (∼2.5-fold) in the growth of MCF-7-shPG relative to MCF-7 cells (Fig. 5A). As additional controls, we also assessed the growth rate of SCC9, MDA-231 and their plakoglobin expressing transfectants SCC9-PG and MDA-231-PG, respectively. In contrast to MCF-7-shPG, the growth rate of SCC9-PG cells was reduced ∼2.5-fold relative to parental SCC9 cells, whereas MDA-231-PG cells showed a 2-fold reduction in growth relative to parental MDA-231 cells (Fig. 5A) These results are consistent with our previous observations in SCC9 and MDA-231 cells [15], [49].

We then used BrdU labeling to verify if the differences observed at the end of the 7-day growth assay among different cell lines with various levels of plakoglobin expression were due to differences in cell proliferation. Cells from various cell lines were plated and allowed to grow for 6 days at which time they were labeled with BrdU for 24 hours and processed for confocal microscopy as described in Materials and Methods. The results showed that SCC9 and MDA-231 cells were highly proliferative as almost all cells displayed BrdU incorporation. In contrast, we detected very little or no BrdU incorporation in the plakoglobin expressing MCF-7, SCC9-PG and MDA-231-PG cells (Fig. 5B), whereas there was significant BrdU incorporation in the plakoglobin knockdown MCF-7-shPG cells (Fig. 5B).

**Figure 6:**
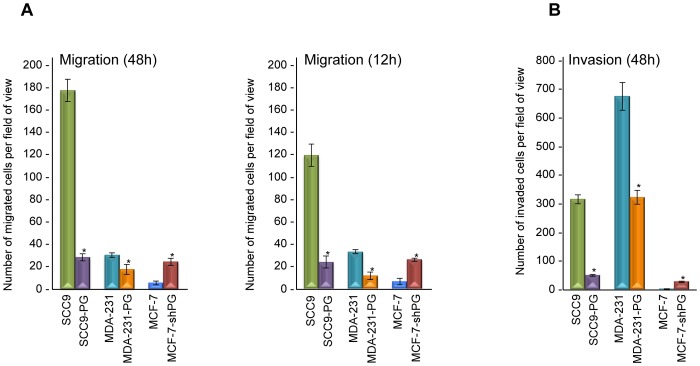
Plakoglobin decreases *in vitro* migration and invasion. **A.** Forty-eight- and twelve-hour Transwell migration assays were performed in triplicates for SCC9, SCC9-PG, MDA-231, MDA-231-PG, MCF-7 and MCF-7-shPG cell lines. The membranes were fixed, stained, cut and mounted on slides and viewed under an inverted microscope. **B.** Forty-eight-hour matrigel invasion assays were performed as described in A using matrigel coated transwell membranes. The number of migrated/invaded cells in five random fields for each membrane were calculated using the ImageJ Cell Counter program and averaged. Histograms represent the average ± SD of the number of migrated/invaded cells for each cell line. *p<0.01). PG, plakoglobin.

The migratory properties of the various cell lines were assessed using transwell chambers. Cells were allowed to migrate through transwell filters for 48 hours, after which the migrated cells were fixed and counted. Consistent with our previous observations, MDA-231-PG cells displayed ∼40% less migration than MDA-231 cells (Fig. 6A; [49]). Similarly, SCC9 cells were approximately 10-fold more migratory than SCC9-PG cells, whereas MCF-7-shPG cells showed a 4-fold increase in migration compared to MCF-7 cells (Fig. 6A). To rule out the possibility that the increased migration in SCC9, MDA-231 and MCF-7-shPG could be due to their higher cell proliferation rate, we repeated the migration assays for 12 hours, since our growth data showed that none of the cell lines had a doubling time less than 24 hours (Fig. 5A). The results of these experiments were consistent with those of the 48 hours assays and showed that SCC9, MDA-231 and MCF-7-shPG cells were considerably more migratory than their plakoglobin-expressing counterparts (SCC9-PG, MDA-231-PG, MCF-7; Fig. 6A).

The invasive properties of the various cell lines were assessed using matrigel-coated transwell chambers. Similar to the migration experiments, cells were allowed to migrate through the matrigel matrix, after which the invaded cells were fixed and counted. These experiments showed that in addition to being more migratory, SCC9, MCF-7-shPG and MDA-231 cells were more invasive than SCC9-PG, MCF-7 and MDA-231-PG cells (approximately 6-, 7- and 2-fold, respectively; Fig. 6B; [49]). Taken together, these results suggest that plakoglobin, regulates the expression of genes involved in cell growth, migration and invasion concurrent with a suppression of *in vitro* migration and invasion.

## Discussion

In the present study, we have further investigated the underlying mechanisms for plakoglobin's role in tumorigenesis and metastasis [also see 14, 49]. Our data showed that plakoglobin associated with the promoter of the oncogenic DNA binding protein SATB1 and downregulated its expression. The decreased expression of *SATB1* following plakoglobin expression was associated with its decreased protein levels and in turn, altered expression of SATB1 target genes with an overall effect of decreased cell growth and *in vitro* migration and invasion. Conversely, knockdown of plakoglobin in MCF-7 cells resulted in the upregulation of SATB1 and increased cell proliferation, migration and invasion.

SATB1's ability to regulate gene expression was initially identified in the thymus, where several studies showed that it was essential for T-cell development and differentiation [24], [30], [50]. These studies demonstrated that SATB1 regulates gene expression by organizing target gene loci into distinct domains/chromatin loop structures and by recruiting different chromatin remodeling enzymes to promote gene expression and T-cell differentiation. Since then, SATB1 has been established as a contributing factor to the development and progression of many different types of cancer, including breast, lung, prostate, colon and ovarian [34]–[42]. SATB1 has also been shown to participate in the epidermis differentiation as SATB1^−/−^ mice showed defects in epidermal differentiation [51]. These defects were associated with the improper activation of genes found within the epidermal differentiation complex locus, to which SATB1 was shown to bind. Other studies have demonstrated that SATB1 regulates the expression of at least 10% of genes in both T-cells and non T-cells, including genes involved in apoptosis, cell-extracellular matrix attachment, cellular metabolism, calcium signaling and the Wnt, Notch, and TGF-β pathways, suggesting that it plays a role in the global regulation of gene expression [32], [52].

SATB1 has been suggested to regulate gene expression in conjunction with β-catenin as part of the Wnt signaling pathway [44], [53]–[54], since during T-cell differentiation, SATB1 associates with and recruits p300/CBP histone acetyltransferase and β-catenin to the promoters of Wnt target genes, resulting in the increased expression of genes such as *IL-2* and *MYC* [44]. SATB1 also associated with the major breakpoint region (mbr) in the 3′-UTR of the *BCL2* gene and promoted the expression of this anti-apoptotic protein, whose expression is also regulated by β-catenin, through the induction of c-Myc and E2F1 [43], [55]–[57]. We previously showed that plakoglobin is also able to regulate the levels of Bcl-2 through the modulation of the signaling activity of β-catenin [58]. The data presented here clearly demonstrates that plakoglobin associates with the *SATB1* promoter and downregulates its expression. Taken together, these observations suggest that plakoglobin may regulate Wnt/β-catenin and SATB1 signaling in multiple ways. First, plakoglobin downregulates the expression of *SATB1*, which would result in the decreased expression of SATB1 target genes. The decreased levels of SATB1 may also alter/reduce β-catenin recruitment to its target promoters and therefore reduce the expression of those genes. Second, nuclear plakoglobin decreases the interaction between β-catenin and TCF and results in inhibition of TCF/β-catenin signaling [13], [58]. Third, expression of physiological levels of plakoglobin results in decreased levels of β-catenin [15], [59]. Finally, plakoglobin associates with and inhibits the expression of the *MYC* promoter [60], a β-catenin and SATB1 target gene [44], [61]–[62].

More recent studies have suggested that SATB1 plays a role in breast tumorigenesis and metastasis. Indeed, SATB1 expression in SATB1 deficient SKBR3 breast cancer cells resulted in increased tumor growth and a more migratory and invasive phenotype that was concurrent with increased expression of tumor/metastasis promoter genes such as c-Abl, Snail, MMP3, TGFβ-1, ErbB2 and decreased expression of tumor/metastasis suppressors including Nm23, Claudin-1, Kiss1, BRMS1, KAI1. Conversely, knockdown of SATB1 in the highly invasive MDA-231 cells had the opposite effect: tumor/metastasis promoting genes were downregulated whereas inhibitors of these processes were upregulated [34].

Plakoglobin also appears to have a role in regulating tumorigenesis and metastasis through the modulation of gene expression. We recently showed that plakoglobin interacts with the transcription factor p53 and regulates the expression of *SFN*, the gene encoding the tumor suppressor 14-3-3σ [14]. Furthermore, we showed that p53-transcriptional activity is enhanced in the presence of plakoglobin and that mutant p53 proteins may, in association with plakoglobin, be functional in regulating their wild-type target genes. In the current study, we have identified *SATB1* as another target gene of plakoglobin and p53, as ChIP experiments clearly demonstrated an association of both proteins with the *SATB1* promoter (Figs. 1–2). However, as opposed to *SFN, SATB1* is negatively regulated by p53 and plakoglobin. While we have shown that plakoglobin and p53 interact with one another [14], whether these interactions are direct or involve other cofactors is not clear and warrants further investigation. Furthermore, although plakoglobin is known to associate with TCF/LEF and regulate gene expression [9], [13], [57], [60], neither the human *SATB1* nor the *NME1* genes contain potential TCF/LEF binding sites, therefore it is likely that plakoglobin-mediated regulation of these genes is independent of TCF/LEF. It was previously shown that p63 is a transcriptional activator of *SATB1* during epidermal differentiation [51], however, to the best of our knowledge, the present work is the first to show that p53 also regulates *SATB1* expression, albeit opposite to p63. What other co-factors are involved in the regulation of p53 and plakoglobin target genes and to what extent these co-factors differ based on whether the complex is activating or repressing gene expression remains unknown and warrants further investigation.

Along with repressing *SATB1* expression, plakoglobin appears to regulate the expression of (at least a subset of) potential SATB1 target genes, including the metastasis suppressor Nm23-H1 [63]. Since its initial discovery, a total of ten Nm23 isoforms (Nm23-H1 to -H10) have been identified in humans, with Nm23-H1 and -H2 being the best studied and characterized [64]–[65]. Nm23-H1 has diverse biological functions including nucleoside diphosphate kinase (NDPK), protein histidine kinase and 3′–5′ exonuclease activities, all of which may potentially contribute to its metastasis suppressor function [66]–[70]. In addition, both Nm23-H1 and -H2 are capable of binding to DNA and regulating gene expression [71]–[77]. Previous studies have shown that exogenous expression of Nm23 in cells lacking its expression resulted not only in decreased migration and invasion, but also in decreased cell proliferation and inhibition of anchorage independent growth [78]–[82]. Furthermore, Nm23 proteins reduced telomerase activity [83] and promoted cell-cell adhesion [84], cell-cycle arrest, and apoptosis [75], as well as DNA-repair following U.V. and ionizing radiation [85]–[86]. These results suggest that Nm23 proteins may also suppress tumor formation in addition to metastasis.

We previously showed that Nm23-H1 mRNA and protein as well as Nm23-H2 protein were upregulated in SCC9-PG cells and that plakoglobin and Nm23 interacted in both the soluble and cytoskeleton-associated pools of cellular proteins [19]. In the present study, we further characterized the role of plakoglobin in the regulation of the *NME1* gene and showed that plakoglobin and p53 associated with the *NME1* promoter and activated its expression (Fig. 3). The association of plakoglobin with the *NME1* promoter is novel and consistent with a previous report that showed decreased Nm23-H1 mRNA levels following plakoglobin knockdown in breast cancer cells [87]. Furthermore, it has been suggested that *NME1* is a transcriptional target of p53, since its mRNA and protein levels were higher in breast and colon carcinoma cell lines expressing active p53 [88]–[89]. These observations are strongly supported by our data, which clearly showed that p53 associated with the *NME1* promoter. Taken together with our previous results, these data suggest that plakoglobin can increase the levels of its potential target genes through different mechanisms, including direct regulation of gene expression (Nm23-H1) and protein-protein interactions that result in increased protein levels (Nm23-H2; [19]).

In addition to *NME1*, we also observed alterations in the mRNA and protein levels of other SATB1 target genes. More specifically, knockdown of plakoglobin in MCF-7 cells resulted in the increased mRNA and protein levels of the tumor/metastasis promoters c-Abl, Snail, ErbB2 and MMP3 and the decreased levels of tumor/metastasis suppressors BRMS1, Kiss1 and Claudin-1. Whether plakoglobin may alter the expression of these SATB1 target genes by altering the expression of SATB1 itself and/or by associating with the promoters of these target genes and promoting/repressing their expression requires further investigation.

We showed that the biological consequence of plakoglobin expression in null/low plakoglobin expressing cells was decreased cell growth and *in vitro* migration and invasion (Figs. 5–6). These results were in agreement with other studies that have previously shown that plakoglobin suppresses these processes and promotes a more “epithelial” phenotype, consistent with its role as a tumor (and metastasis) suppressor [15], [20], [49], [87].

Increasing evidence suggests that plakoglobin regulates tumorigenesis independent of its cell-cell adhesion function. Plakoglobin regulates the expression of genes such as *MYC, DSC2* and *SFN* [14], [60], [90] and also suppresses Ras-mediated oncogenesis through increased HDAC4 mRNA levels [91]. In addition to regulation of gene expression, plakoglobin has been shown to act as a tumor/metastasis suppressor by modulating Rho, Fibronectin and Vitronectin-dependent Src signaling [21], [92].

Our findings are significant in that they clearly point to a role of plakoglobin in regulating a variety of genes that are involved in tumor progression and metastasis. Our data also suggests that plakoglobin may regulate a number of genes under normal cellular conditions (i.e. in the absence of cell stress or activation of different growth pathways), implying that plakoglobin may be a “basal” and more global type of regulator of gene expression. As such, our results have larger implications in that plakoglobin may have a potential as a new therapeutic target for the treatment of various cancers.
